# Awareness and Knowledge of Gastric Cancer in the General Population of Jeddah, Saudi Arabia: A Cross-Sectional Study

**DOI:** 10.3390/healthcare14121743

**Published:** 2026-06-17

**Authors:** Ashraf A. Maghrabi, Moaz W. Abulfaraj, Wisam Jamal, Rayan J. Alotaibi, Omar M. Saggaf, Suha Kaaki, Amna Hussein Alkhaldi, Emad Aljahdali, Murad M. Aljiffry

**Affiliations:** 1 Department of Surgery, Faculty of Medicine, King Abdulaziz University, Jeddah 21589, Saudi Arabia; aammaghrabi@kau.edu.sa (A.A.M.); maljefri@kau.edu.sa (M.M.A.); 2 Department of Surgery, Alsalama Hospital, Jeddah 23611, Saudi Arabia; 3 Department of Surgery, Faculty of Medicine, University of Jeddah, Jeddah 21589, Saudi Arabia; 4 Faculty of Medicine, King Abdulaziz University, Jeddah 21589, Saudi Arabia; rraan-422@hotmail.com (R.J.A.); o.saggaf@outlook.com (O.M.S.); ahmalkhaldi@kau.edu.sa (A.H.A.); 5 Department of Surgery, School of Medicine, King Saud University, Riyadh 12584, Saudi Arabia; dr.kaaki@gmail.com; 6 Department of Medicine, Faculty of Medicine, King Abdulaziz University, Jeddah 21589, Saudi Arabia; ealjahdli@gmail.com

**Keywords:** gastric cancer, awareness, risk factors, symptom recognition, Saudi Arabia, cross-sectional study, cancer prevention

## Abstract

**Highlights:**

**What are the main findings?**

- Public awareness of gastric cancer in Jeddah was suboptimal (mean score 51.2%), with knowledge of risk factors and recognition of symptoms representing the weakest domains.
- Awareness levels were significantly associated with occupation, education, age, and personal experience with gastric cancer; healthcare workers achieved substantially higher scores than all other groups.

**What are the implications of the main findings?**

- Targeted public health interventions should prioritize alarm symptom recognition, Helicobacter pylori awareness, and dietary risk factors.
- Primary care settings, community channels, and outreach programs targeting first-degree relatives of patients with gastric cancer may represent the most actionable delivery routes.

**Abstract:**

**Background/Objectives**: Gastric cancer is a major cause of morbidity and mortality in Saudi Arabia, where late-stage presentation is common. Public awareness of risk factors and warning symptoms is essential for early detection. This study assessed gastric cancer knowledge and its demographic correlates among adults in Jeddah. **Methods**: A cross-sectional survey of 1400 adults was conducted between August and October 2025 using a structured 45-item questionnaire covering 37 knowledge items across four domains. Analyses used independent *t*-tests, one-way ANOVA, chi-squared tests, and multivariable linear regression. **Results**: The mean age was 31.34 ± 10.18 years; 53.8% were women. The mean total knowledge score was 18.94 ± 7.34/37 (51.2%). Domain scores were risk factors 4.95 ± 2.04/11 (45.0%), symptoms 4.54 ± 2.87/10 (45.4%), prevention 4.91 ± 2.09/10 (49.1%), and management 2.99 ± 1.95/6 (49.8%). Knowledge was associated with occupation (*p* < 0.001), with healthcare workers scoring highest (25.97 ± 5.56). Higher knowledge was also associated with education (*p* < 0.001), age (*p* = 0.003), and family or friend history of gastric cancer (*p* = 0.003), but not sex (*p* = 0.39). In multivariable analysis, educational attainment (β = 0.30) and healthcare-provider occupation (β = 0.23) were the strongest independent correlates of knowledge (both *p* < 0.001). Only 34% of respondents identified *Helicobacter pylori* as a risk factor. **Conclusions**: Awareness of gastric cancer in Jeddah is suboptimal, particularly for risk factors and symptom recognition. Targeted interventions addressing risk factors, alarm symptoms, and dietary risks—delivered through primary care and community channels—may improve awareness and support earlier detection.

## 1. Introduction

Gastric cancer (GC) is the fifth most commonly diagnosed cancer and the fifth leading cause of cancer-related death worldwide, with an estimated 968,000 new cases and 660,000 deaths in 2022, accounting for 4.9% and 6.8% of global totals, respectively [1,2]. Although its incidence has declined in many regions over recent decades, largely due to improved sanitation, food preservation, dietary changes, and, most importantly, reduced *Helicobacter pylori* infection prevalence [2,3], the global burden remains substantial. Projections suggest that annual incidence will reach approximately 1.45 million cases by 2050, driven primarily by population growth and aging [3,4].

In Saudi Arabia, the age-standardized incidence rate (ASIR) of GC is estimated at 2.7 per 100,000 (both sexes, all ages), based on GLOBOCAN 2020 and 2022 data, ranking 15th among cancers nationally, with an age-standardized mortality rate (ASMR) of 2.1 per 100,000 [5,6]. Registry-based analyses from the Saudi Cancer Registry (2004–2017) indicate a stable to slightly declining ASIR, with the highest rates observed in Riyadh, Najran, and the Eastern Region, and a male-to-female ratio of approximately 2:1 [6]. These rates are lower than those in neighboring Gulf countries—Oman (8.0), Qatar (5.2), Bahrain (4.8), and the United Arab Emirates (4.4) per 100,000 [6,7]. Despite this comparatively low incidence, GC remains a key public health concern in Saudi Arabia because most patients are diagnosed at advanced TNM stages [8,9]. Emerging evidence of early-onset disease among younger individuals, likely linked to lifestyle and dietary changes, further heightens this concern [10].

Established risk factors for GC include chronic *H. pylori* infection—responsible for approximately 76% of lifetime non-cardia cases globally and affecting roughly half of the global population [4,11,12]—as well as dietary factors such as high salt intake and frequent consumption of smoked, pickled, and nitrite-containing processed foods [13,14], tobacco and waterpipe smoking [15], obesity, and a family history of GC [16]. The mortality gap between high- and low-incidence regions is largely attributable to organized endoscopic screening in East Asia [17,18]. In the absence of a national screening program in Saudi Arabia—given limited cost-effectiveness at the current ASIR—public awareness of modifiable risk factors and warning symptoms is critical to promoting risk reduction, increasing *H. pylori* testing uptake, and encouraging earlier presentation [19,20].

Several recent Saudi studies have examined public awareness of GC. Alghamdi et al. (2023) reported suboptimal knowledge among 426 adults in Al-Baha, with alcohol use, smoking, and family history being the most commonly recognized risk factors [21]. A nationwide knowledge, attitudes, and practices (KAP) study by Alzahrani et al. (2024), involving 2541 respondents, found “good” knowledge in 59.4% but identified persistent gaps in understanding the transmission and contagiousness of *H. pylori* [22]. Jamal et al. (2026) reported that although 81.8% of 451 respondents were aware of cancer screening in general, knowledge of specific programs remained limited [23]. Similarly, Alameer et al. (2024) documented suboptimal overall cancer awareness in Bisha Province [24]. A 2025 review of gastrointestinal cancer trends in Saudi Arabia highlighted low public awareness as a priority for Vision 2030 health reform [25]. However, Jeddah—Saudi Arabia’s second-largest city and largest urban center on the western coast—has not been examined in a large-sample study of GC awareness.

The Ministry of Health has historically placed less emphasis on GC education than on other malignancies, such as breast and lung cancer, and no national GC screening program exists [26]. Accordingly, this study aimed to (i) quantify public knowledge of GC across four predefined domains among adults in Jeddah, (ii) identify specific knowledge gaps amenable to targeted interventions, and (iii) examine sociodemographic factors associated with knowledge levels to inform public health campaigns aligned with Saudi Vision 2030.

## 2. Materials and Methods

### 2.1. Study Design and Setting

This cross-sectional community survey was conducted among adult residents of Jeddah, Saudi Arabia, between August and October 2025. Reporting followed the Strengthening the Reporting of Observational Studies in Epidemiology (STROBE) guidelines [27], with the completed checklist provided as Supplementary Material S3. The study was approved by the Institutional Review Board of King Abdulaziz University, Faculty of Medicine, Jeddah, on 16 June 2025, and conducted in accordance with the 1964 Declaration of Helsinki and its later amendments. Written or electronic informed consent was obtained from all participants prior to enrollment (Supplementary Material S1).

### 2.2. Sample and Recruitment

A total of 1400 residents of Jeddah were recruited using convenience sampling at public malls and other public venues, supplemented by an electronic questionnaire distributed via WhatsApp. Both in-person and online responses were collected concurrently during the same period using an identical instrument. Participation was voluntary and non-incentivized, and the survey link could be shared with others, consistent with a non-probability convenience and snowball sampling approach. Inclusion criteria were age ≥ 18 years and residence in Jeddah. The sample comprised workers, non-workers, students, and healthcare providers across a broad range of ages and educational backgrounds, with no additional exclusion criteria.

### 2.3. Data Collection Instrument

The 45-item questionnaire was adapted from validated instruments used in previous GC awareness studies [21,28] and underwent face and content validation by surgical faculty at King Abdulaziz University Hospital and other expert clinicians. The instrument was administered in Arabic, with back-translation to English performed to ensure fidelity to the original items. It comprised 8 demographic items (age, sex, education, occupation, personal history of gastrointestinal disease, personal history of GC, family or friend history of GC, and source of health information) and 37 knowledge items across 4 domains: risk factors (11 items), symptoms and signs (10 items), prevention (10 items), and management (6 items). Knowledge items were answered using a true/false/don’t know format, with one point assigned for each correct response (maximum score: 37). A pilot test in a convenience sample (not included in the final analysis) confirmed item clarity and acceptability prior to full deployment.

### 2.4. Statistical Analysis

Analyses were conducted using IBM SPSS Statistics, version 30 (IBM Corp., Armonk, NY, USA). Continuous variables are presented as means ± standard deviations and categorical variables as frequencies and percentages. Between-group differences in mean knowledge scores were assessed using the independent-samples *t*-test for two groups and one-way analysis of variance (ANOVA) with Tukey post hoc testing for multiple group comparisons. Associations between categorical variables were evaluated using the chi-squared test. All tests were two-tailed, and a *p*-value < 0.05 was considered statistically significant. Before inferential testing, the distribution of the total knowledge score was assessed and found to be approximately normal, with skewness and kurtosis values within ±1, supporting the use of parametric tests. To identify factors independently associated with the total knowledge score, a multivariable linear regression model was fitted, with the total score as the dependent variable and occupation (reference: non-worker), age group (reference: 26–35 years), educational attainment (entered as an ordinal variable), sex (reference: male), and having a family member or friend with GC as independent variables. Unstandardized coefficients (B) with 95% confidence intervals (CIs), standardized coefficients (β), and the coefficient of determination (R^2^) are reported.

## 3. Results

### 3.1. Participant Characteristics

A total of 1400 respondents completed the survey. The mean age was 31.34 ± 10.18 years (range, 18–66 years). Of the participants, 753 (53.8%) were women and 647 (46.2%) were men. By occupation, 619 (44.2%) were workers, 388 (27.7%) were non-workers, 284 (20.3%) were students, and 109 (7.8%) were healthcare providers (including physicians and nurses). In addition, 350 respondents (25.0%) reported having a family member or friend diagnosed with GC, and 25 (1.8%) reported a personal history of GC. Detailed demographic characteristics are presented in Table 1.

### 3.2. Overall Knowledge Score

The mean total knowledge score was 18.94 ± 7.34 out of 37 (51.2%). Overall, 696 respondents (49.7%) scored below the mean, while 704 (50.3%) scored at or above the mean score.

### 3.3. Knowledge Scores by Domain

**Figure 1:**
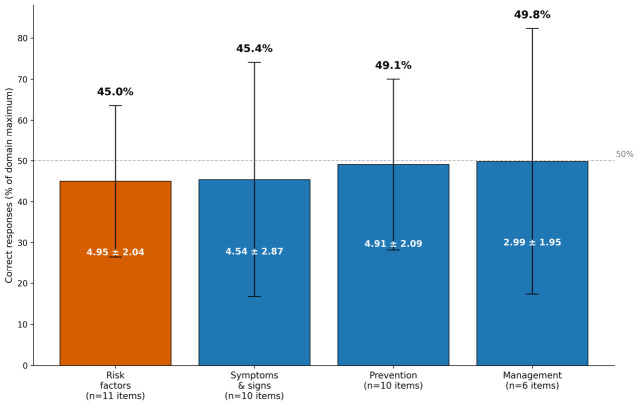
Knowledge scores by domain (*n* = 1400). Error bars indicate ± 1 standard deviation (SD).

**Figure 2:**
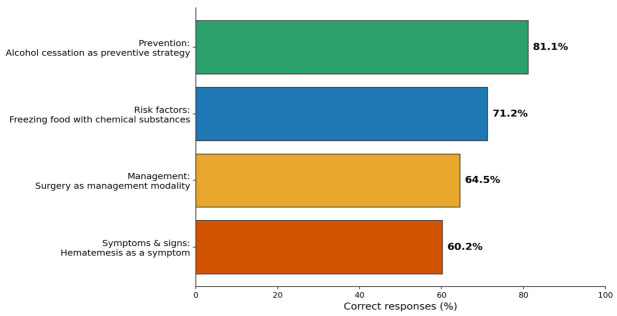
Highest correctly recognized item within each knowledge domain (*n* = 1400).

Domain-level mean knowledge scores showed modest variation across the four content areas. Participants performed best in the management domain (2.99 ± 1.95/6; 49.8%), followed closely by prevention (4.91 ± 2.09/10; 49.1%), while the lowest performance was observed in the symptoms and signs domain (4.54 ± 2.87/10; 45.4%) and the risk factors domain (4.95 ± 2.04/11; 45.0%) (Figure 1). The most frequently correctly identified item in each domain (Figure 2) was recognition of “freezing food using chemical substances” as a risk factor for gastric carcinoma (71.2%) in the risk factors domain, hematemesis as a symptom of gastric carcinoma (60.2%) in the symptoms domain, alcohol cessation as a preventive strategy (81.1%) in the prevention domain, and surgery as a management option for gastric carcinoma (64.5%) in the management domain.

### 3.4. Knowledge by Occupation

**Figure 3:**
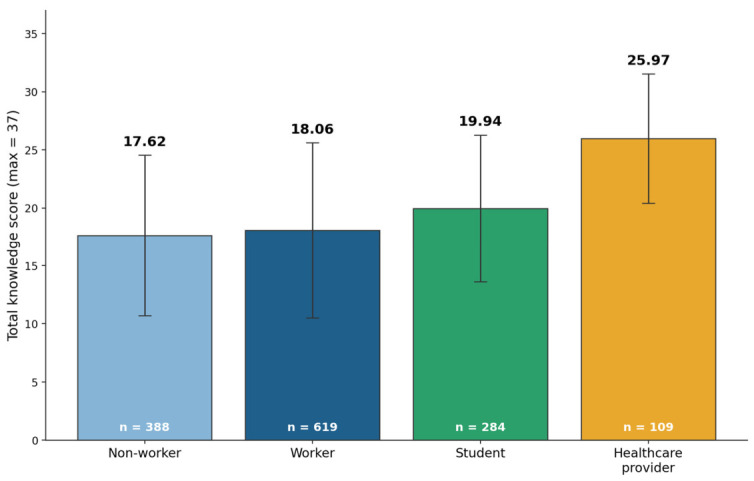
Mean total knowledge score by occupation (*n* = 1400). Error bars indicate ±1 standard deviation (SD).

Occupation was strongly associated with the total knowledge score (one-way ANOVA, *p* < 0.001). Healthcare providers achieved substantially higher mean scores than all other occupational groups across all knowledge domains (Table 2, Figure 3). Students scored higher than both workers and non-workers, while non-workers had the lowest mean score among the non-healthcare groups.

### 3.5. Knowledge by Age Group and Other Demographics

**Figure 4:**
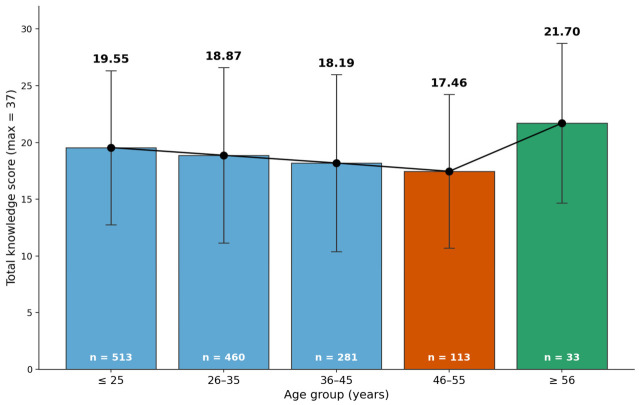
Total knowledge score by age group (*n* = 1400). Error bars indicate ± 1 standard deviation (SD).

Knowledge scores varied significantly across age groups (one-way ANOVA, *p* = 0.003; Figure 4). The ≥56-year group had the highest mean score (21.70 ± 7.04), followed by the ≤25-year group (19.55 ± 6.78), while the 46–55-year group had the lowest score (17.46 ± 6.77) (Table 3). Sex was not significantly associated with knowledge (men: 18.78 ± 7.55; women: 19.07 ± 7.18; *p* = 0.39). Participants with a family member or friend diagnosed with GC had higher mean knowledge scores than those without such a history (20.07 ± 7.00 vs. 18.56 ± 7.42; *p* = 0.003). The 25 respondents with a personal history of GC had a mean score of 18.40 ± 6.08, comparable to the overall sample mean. Educational attainment was also significantly associated with knowledge (*p* < 0.001), with mean scores increasing progressively from 15.12 ± 6.65 among participants with less than a high school education to 23.41 ± 6.78 among those with postgraduate degrees (Table 4). The complete set of associations between sociodemographic factors, excluding age and occupation, and total knowledge score is presented in Table 4.

At the item level, recognition of *H. pylori* infection as a risk factor for GC was low. Only 34% of respondents correctly identified it, making it one of the least recognized risk factors despite its well-established role as the primary modifiable cause of non-cardia GC.

### 3.6. Factors Independently Associated with Knowledge (Multivariable Analysis)

In the multivariable linear regression model (Table 5), educational attainment was the strongest independent correlate of total knowledge (B = 1.45 per level; 95% CI, 1.20–1.70; β = 0.30; *p* < 0.001), followed by healthcare-provider occupation relative to non-workers (B = 5.75; 95% CI, 4.37–7.13; β = 0.23; *p* < 0.001). Belonging to the ≥56-year age group (B = 3.21; 95% CI, 0.96–5.46; *p* = 0.005) and having a family member or friend with GC remained independently associated with higher scores after adjustment (B = 0.93; 95% CI, 0.12–1.74; *p* = 0.025). The apparent advantage of the youngest group (≤25 years) attenuated to borderline significance (*p* = 0.058), indicating that the higher unadjusted score was largely explained by educational attainment and student status. Sex (*p* = 0.273) and worker or student status were not independently associated with knowledge.

The model explained a modest proportion of the variance (R^2^ = 0.146; adjusted R^2^ = 0.140; F(10, 1389) = 23.83; *p* < 0.001), suggesting that most individual variation in knowledge was attributable to factors not captured by demographic characteristics included in the model.

## 4. Discussion

This study provides the first large-sample assessment of GC awareness among adults in the western region of Saudi Arabia. Four main findings emerged. First, overall knowledge was suboptimal, with a mean score of 51.2% of the maximum, and the risk factors (45.0%) and symptoms and signs (45.4%) domains representing the weakest areas. Second, educational attainment and healthcare-provider occupation were the factors most strongly and independently associated with knowledge in multivariable analysis. Healthcare workers demonstrated substantially higher knowledge than all other occupational groups, although part of this difference was attributable to differences in educational level. Third, older age (≥56 years) and prior exposure to GC through a family member or friend were independently associated with higher knowledge, whereas sex showed no significant association. Fourth, recognition of risk factors and warning symptoms consistently lagged behind knowledge of prevention and management across all occupational groups, highlighting a key target for future public health interventions.

Interpreting the model as a whole, educational attainment (β = 0.30) and healthcare-provider occupation (β = 0.23) were the only strong independent correlates of knowledge. Worker and student status lost significance once education and age were accounted for, indicating that the crude occupational differences largely reflected educational composition, whereas the 5.75-point advantage healthcare workers retained after adjustment points to a profession-specific training effect. The model nonetheless explained only a modest share of the variance (*R*^2^ = 0.146), implying that most individual variation in knowledge reflects unmeasured factors—such as health-seeking behavior and media exposure—rather than the measured demographics, and that awareness efforts will require broad-reach campaigns rather than demographic targeting alone.

Our overall mean score of 51.2% is broadly comparable with previous Saudi studies. Earlier work by Ravichandran et al. (2010) reported limited public cancer knowledge in the Riyadh region [29], and more recent Alghamdi et al. (2023) reported suboptimal GC knowledge in Al-Baha [21], while Alzahrani et al. (2024) found “good” *H. pylori* knowledge in 59.4% of a nationwide sample of 2541 respondents using a 5-item instrument [22]. Although instruments and cutoff thresholds differ across studies, these findings collectively suggest that GC awareness in Saudi Arabia remains suboptimal and variable across regions. The marked advantage observed among healthcare workers is consistent with findings from similar studies in Iran [28] and other settings.

The U-shaped relationship with age—where both the youngest (≤25 years) and oldest (≥56 years) groups scored higher than middle-aged respondents—is an unexpected but plausible pattern. The higher scores among younger respondents may reflect ongoing formal education, as students were concentrated in this age group. Consistent with this, the higher score observed in the youngest group attenuated to borderline significance after adjustment for education and occupation (*p* = 0.058), indicating that it was largely driven by these factors rather than age itself. By contrast, the ≥56-year group remained independently associated with higher knowledge in the multivariable model (*p* = 0.005); however, this group was small (*n* = 33), and the corresponding estimate is therefore imprecise (95% CI, 0.96–5.46). This older group may have greater cumulative exposure to personal or family illness and cancer-related health messaging, whereas the 46–55-year group may reflect competing work and family responsibilities, potentially limiting exposure to health education content, although this hypothesis warrants further investigation. This pattern contrasts with a similar Iranian study, which reported a middle-aged peak (46–55 years) [28], and suggests that Saudi public health campaigns may need to be more explicitly age-stratified rather than primarily targeting middle-aged adults.

The finding that respondents with a family member or friend diagnosed with GC had significantly higher knowledge (20.07 vs. 18.56; *p* = 0.003) is consistent with a UK population-based study reporting similar associations [30]. This aligns with evidence that cancer experience within an individual’s social network promotes information-seeking behavior and improved knowledge retention. In contrast, the 25 respondents with a personal history of GC had a mean score (18.40) comparable to the overall sample mean (*p* = 0.92). Given the small size of this subgroup, the study was underpowered to detect meaningful differences, and this finding should be interpreted cautiously rather than as evidence of true equivalence in knowledge. The wide uncertainty around this estimate limits firm conclusions. Nonetheless, it may indicate an opportunity to strengthen patient education and survivorship counseling following diagnosis.

The domain-specific findings are informative. In the risk factor domain—the lowest-performing area overall (45.0%)—the highest correctly identified item (71.2%) concerned chemical food preservation, suggesting that public messaging on food additives may be relatively effective whereas other risk factors remain poorly recognized. In the symptoms domain, hematemesis was the best-recognized item (60.2%), and no symptom achieved recognition levels comparable to leading items in other domains, confirming symptom awareness as among the weakest areas. In the prevention domain, alcohol cessation was most frequently identified (81.1%), likely reflecting cultural and religious norms in Saudi Arabia rather than specific cancer-prevention knowledge. In the management domain, surgery was correctly identified by 64.5% of respondents as a treatment modality. Collectively, these patterns indicate that future educational efforts should prioritize risk factor awareness and recognition of symptoms—particularly alarm features beyond hematemesis, including unintentional weight loss, dysphagia, persistent epigastric pain, early satiety, and iron-deficiency anemia, as these domains represent the greatest opportunity to improve early presentation.

These findings have practical implications for public health and clinical practice. The observed occupational gradient, with healthcare workers substantially better informed than the general public, supports the use of primary care encounters as opportunistic settings for GC education. Similarly, the association between family history and higher knowledge supports targeted outreach to relatives of patients through oncology and surgical clinics. Despite Saudi Arabia’s comparatively low regional incidence, the persistence of late-stage presentation justifies continued investment in awareness strategies.

Critically, although incidence in Saudi Arabia is among the lowest globally, outcomes for individuals diagnosed with GC remain poor, and this pattern extends across the Gulf region and the broader Arab region. Aoude et al. (2022), in a systematic review of GC in the Arab world, reported a 5-year overall survival of only 16.5% in Oman and 21.1% in Jordan [7]. UAE registry data similarly indicate that most GC cases (approximately 71% of staged cases in 2017) present at advanced stages, with the remainder predominantly classified as unknown stage, reflecting limitations in registry documentation [31]. Saudi single-center cohorts mirror these findings, with 5-year survival rates ranging from 19.6% (King Khalid Hospital, Jeddah) to 37.4% (Najran), and up to 74.6% of patients presenting at TNM stage IV [8,9].

By contrast, 5-year survival rates reach 78–81% in Japan and the Republic of Korea, where organized endoscopic screening programs enable early detection, and survival for localized disease exceeds 95% [17,18,32]. The mortality-to-incidence (M/I) ratio of approximately 0.78 in Saudi Arabia (ASMR 2.1/ASIR 2.7 per 100,000) [5,6] is therefore higher than those reported for high-incidence East Asian countries, despite a substantially lower disease burden. This disparity is driven primarily by stage at presentation rather than tumor biology and is similarly observed across Saudi Arabia’s Gulf neighbors. Table 6 summarizes these international comparisons.

Overall, this survival gap reinforces the central rationale for the present study: in a setting without a national screening program, public awareness of warning symptoms and risk factors represents a key modifiable determinant of stage at diagnosis and, consequently, survival.

### 4.1. Implications for the Saudi Vision 2030 Public Health Strategy

The awareness gaps identified in this study align with three key Saudi Vision 2030 health sector priorities: strengthening preventive care, reducing the burden of non-communicable diseases through lifestyle modification, and improving population health literacy [26]. The most cost-effective intervention suggested by our findings is an integrated public health campaign combining *H. pylori* “test-and-treat” messaging—supported by meta-analytic evidence that eradication therapy reduces GC incidence [33]—with education on dietary risk factors and alarm symptoms. Such an approach could be delivered through primary care encounters, social media platforms—given the relatively young age of our sample (mean age, 31.3 years)—and, importantly, targeted outreach to first-degree relatives of patients with GC via oncology and surgical clinics, reflecting the observed family-history gradient. This emphasis on awareness and selective, risk-stratified case finding rather than population-wide endoscopic screening is consistent with evidence suggesting that organized screening is not cost-effective in low-incidence settings. In such contexts, a targeted approach focused on high-risk groups—including individuals with a family history of GC—is recommended instead [34]. A logical next step would be a cluster-randomized trial in Jeddah primary care settings evaluating a structured GC awareness module versus usual care, with outcomes including knowledge improvement and symptom-driven healthcare-seeking behavior.

### 4.2. Strengths and Limitations

Strengths of this study include a large sample size (*n* = 1400), a multidomain questionnaire assessing risk factors, symptoms, prevention, and management in parallel—an approach rarely used in Saudi cancer awareness research, which is often limited to single-domain assessments—and adherence to STROBE reporting guidelines (Supplementary Material S3). Several limitations should be acknowledged. First, recruitment was limited to Jeddah, a city with multiple universities and relatively higher socioeconomic and educational levels, which may have led to an overestimation of knowledge and may limit the generalizability of these findings to the broader Saudi population. Second, despite the use of both in-person and electronic recruitment strategies across public venues, individuals of lower socioeconomic status are likely underrepresented. Third, the true/false/don’t know format may have inflated correct responses due to guessing, particularly for items with inferable answers. Fourth, the cross-sectional design precludes causal inference regarding associations between demographic factors and knowledge. Fifth, although the Arabic questionnaire underwent expert content validation, full psychometric testing (e.g., test–retest reliability and confirmatory factor analysis) was not performed. Sixth, items specifically addressing *H. pylori* awareness, dietary salt intake, and nitrite-containing processed meats were not sufficiently granular for direct comparison with more recent studies; future research should include dedicated items on *H. pylori*. Seventh, multiple between-group comparisons were conducted across several demographic variables without formal adjustment for multiple testing. Although the multivariable model helps mitigate this concern for the primary associations, the descriptive subgroup comparisons should be interpreted with caution. Eighth, the number of individuals approached was not recorded; therefore, a formal response rate could not be calculated, and non-response bias cannot be excluded. Ninth, a priori sample size or power calculation was also not performed; however, the large sample size (*n* = 1400) provided adequate precision for the primary descriptive estimates and supported the multivariable analysis. Finally, as with all self-reported knowledge surveys, responses may be influenced by social desirability bias, particularly for culturally sensitive items.

## 5. Conclusions

GC knowledge among adults in Jeddah is suboptimal, with a mean score of 51.2% of the maximum and risk factor knowledge (45.0%) and symptom recognition (45.4%) representing the weakest domains. Healthcare workers demonstrated substantially higher knowledge than other occupational groups, highlighting their potential role in delivering community-based education. Older age (≥56 years) and having a family member or friend with GC were associated with higher knowledge, whereas sex showed no significant association. Targeted Saudi Vision 2030-aligned public health interventions emphasizing alarm symptom recognition, *H. pylori* awareness, and dietary risk factors—delivered through primary care, social media, and outreach to relatives of diagnosed patients—represent the most actionable strategies supported by these findings. Future research should prioritize cluster-randomized trials evaluating such interventions, with outcomes including knowledge improvement and earlier healthcare-seeking in response to symptoms.

## Figures and Tables

**Table 1:** Sociodemographic characteristics of survey participants (*n* = 1400).

Characteristic	*n*	%
Sex		
Women	753	53.8
Men	647	46.2
Age group, years		
≤25	513	36.6
26–35	460	32.9
36–45	281	20.1
46–55	113	8.1
≥56	33	2.4
Occupation		
Worker	619	44.2
Non-worker	388	27.7
Student	284	20.3
Healthcare provider	109	7.8
Personal connection to gastric cancer		
Family member or friend with GC	350	25.0
Personal history of GC	25	1.8

GC, gastric cancer. Mean age was 31.34 ± 10.18 years (range 18–66). Percentages are calculated from *n* = 1400.

**Table 2:** Knowledge scores by occupation (*n* = 1400).

Occupation	*n*	Total Score	Risk Factors	Symptoms and Signs	Prevention	Management
Worker	619	18.06 ± 7.55	4.78 ± 2.10	4.11 ± 2.88	4.77 ± 2.22	2.89 ± 1.99
Non-worker	388	17.62 ± 6.93	4.49 ± 1.96	4.11 ± 2.64	4.79 ± 2.07	2.73 ± 1.90
Student	284	19.94 ± 6.34	5.37 ± 1.77	4.94 ± 2.72	5.07 ± 1.80	3.00 ± 1.83
Healthcare provider	109	25.97 ± 5.56	6.47 ± 1.76	7.45 ± 2.10	5.77 ± 1.86	4.50 ± 1.53
Total	1400	18.94 ± 7.34	4.95 ± 2.04	4.54 ± 2.87	4.91 ± 2.09	2.99 ± 1.95

Values are mean ± standard deviation (SD). One-way ANOVA for occupation versus total score: *p* < 0.001. Maximum possible scores by domain: risk factors (11), symptoms and signs (10), prevention (10), management (6); total (37). Nominal *p*-values are reported; no adjustment for multiple comparisons was performed.

**Table 3:** Total knowledge scores by age group (*n* = 1400).

Age Category	*n*	Mean ± SD
≤25 years	513	19.55 ± 6.78
26–35 years	460	18.87 ± 7.72
36–45 years	281	18.19 ± 7.79
46–55 years	113	17.46 ± 6.77
≥56 years	33	21.70 ± 7.04
Total	1400	18.94 ± 7.34

One-way ANOVA: *p* = 0.003. Post hoc Tukey analysis showed significantly higher scores in the ≥56-year group compared with the 46–55-year group (*p* < 0.01). Nominal *p*-values are reported; no adjustment for multiple comparisons was performed.

**Table 4:** Total knowledge scores according to sex, educational attainment, and history of gastric cancer (*n* = 1400).

Variable	*n*	Mean ± SD	*p*-Value
Sex			0.39
Women	753	19.07 ± 7.18	
Men	647	18.78 ± 7.55	
Educational attainment			<0.001
Less than high school	180	15.12 ± 6.65	
High school	418	17.05 ± 6.82	
Bachelor’s degree/Diploma	652	19.85 ± 7.12	
Postgraduate (Master’s/PhD)	150	23.41 ± 6.78	
Family member or friend with GC			0.003
Yes	350	20.07 ± 7.00	
No	1050	18.56 ± 7.42	
Personal history of GC			0.92
Yes	25	18.40 ± 6.08	
No	1375	18.95 ± 7.36	

GC, gastric cancer; SD, standard deviation. *p*-values are from independent-samples *t*-tests for binary variables (sex, family/personal history) and one-way ANOVA for educational attainment. Maximum total knowledge score = 37. The personal-history subgroup comprised only 25 participants and is underpowered; this comparison should be interpreted with caution. Nominal *p*-values are reported; no adjustment for multiple comparisons was performed.

**Table 5:** Multivariable linear regression of factors associated with total gastric cancer knowledge score (*n* = 1400).

Variable	B	SE	β	t	*p*-Value	95% CI Lower	95% CI Upper
Constant	15.84	0.58	—	27.47	<0.001	14.71	16.97
Occupation (ref: Non-worker)							
Worker	−0.30	0.43	−0.02	−0.71	0.479	−1.14	0.53
Student	0.09	0.51	0.00	0.17	0.868	−0.92	1.09
Healthcare provider	5.75	0.70	0.23	8.18	<0.001	4.37	7.13
Age group (ref: 26–35 years)							
≤25 years	0.80	0.42	0.06	1.89	0.058	−0.03	1.63
36–45 years	0.80	0.50	0.05	1.60	0.110	−0.18	1.77
46–55 years	0.68	0.73	0.03	0.93	0.352	−0.75	2.12
≥56 years	3.21	1.15	0.09	2.80	0.005	0.96	5.46
Educational attainment (per level)	1.45	0.13	0.30	11.49	<0.001	1.20	1.70
Sex (ref: male)—female	−0.39	0.36	−0.03	−1.10	0.273	−1.09	0.31
Family member/friend with GC (Yes)	0.93	0.41	0.07	2.24	0.025	0.12	1.74

B, unstandardized regression coefficient; SE, standard error; β, standardized coefficient; CI, confidence interval; GC, gastric cancer. Dependent variable: total knowledge score (maximum 37). Reference categories: non-worker (occupation), 26–35 years (age), male (sex), and no family member or friend with GC. Educational attainment was entered as an ordinal variable. Model: R^2^ = 0.146; adjusted R^2^ = 0.140; F(10, 1389) = 23.83; *p* < 0.001.

**Table 6:** International comparison of gastric cancer epidemiology, stage at presentation, and survival outcomes.

Region/Country	ASIR (per 100,000)	ASMR (per 100,000)	Stage IV at Presentation (%)	5-Year Survival (%)	Source
World	9.2	6.1	—	20–30	GLOBOCAN 2022; Thrift 2023 [1,3]
East Asia (male)	22.4	10.3	—	—	GLOBOCAN 2022 [1]
Japan	27.6	9.6	~15	81.0	GLOBOCAN 2022; Hamashima 2018 [1,17]
Republic of Korea	27.0	6.9	~11	78.4	GLOBOCAN 2022; Suh 2020 [1,18]
Europe	8.4	6.0	~40	~25	GLOBOCAN 2022 [1]
United States	5.5	2.4	~35	45.0	GLOBOCAN 2022; Matsuda 2011 [1,32]
Oman	8.0	6.4	—	16.5	Aoude 2022 [7]
Jordan	—	—	—	21.1	Aoude 2022 [7]
Qatar	5.2	3.7	—	—	Aoude 2022/IARC [7]
Bahrain	4.8	3.4	—	—	Aoude 2022/IARC [7]
United Arab Emirates	4.4	3.1	~71	—	Aoude 2022/IARC; UAE Oncology Task Force 2020 [7,31]
Saudi Arabia	2.7	2.1	~50–75	19.6–37.4	IARC; Alghamdi 2023; Badheeb 2025; Aldhafeeri 2021 [5,6,8,9]
Kuwait	2.7	2.0	—	—	GLOBOCAN 2020 [7]

ASIR, age-standardized incidence rate; ASMR, age-standardized mortality rate. Both sexes and all ages unless otherwise stated. The East Asia ASIR is male-specific, derived from World Cancer Report/IARC regional summaries. Stage IV proportions and 5-year survival estimates are derived from country-level cohort and registry studies; where ranges are reported, these reflect variation across single-center series. The UAE estimate (~71%) is based on 2017 UAE registry data for staged cases (30 advanced of 42 staged cases). In the same year, 55% of cases were classified as unknown stage, highlighting the broader limitations in cancer registry completeness in the region. Em dashes (—) indicate that population-based data were unavailable for the corresponding variable at the time of writing.

## Data Availability

The data presented in this study are available on reasonable request from the corresponding author. The data are not publicly available due to privacy considerations associated with participant-level survey responses.

## Supplementary Materials

The following supporting information can be downloaded at https://www.mdpi.com/article/10.3390/healthcare14121743/s1. Supplementary Material S1: Standardized Recruitment Script. Supplementary Material S2: Full 45-Item Questionnaire (English and Arabic versions). Supplementary Material S3: Completed STROBE Checklist for Cross-Sectional Studies.

## Abbreviations

The following abbreviations are used in this manuscript:

GC	Gastric cancer
ASIR	Age-standardized incidence rate
ASMR	Age-standardized mortality rate
ANOVA	Analysis of variance
SD	Standard deviation
STROBE	Strengthening the Reporting of Observational Studies in Epidemiology
KAP	Knowledge, attitudes, and practices
GLOBOCAN	Global Cancer Observatory (IARC global cancer statistics database)
IARC	International Agency for Research on Cancer
WAQF	King Abdulaziz University Endowment
DSR	Deanship of Scientific Research
*H. pylori*	*Helicobacter pylori*
