# Supplementary material for: Awareness and Knowledge of Gastric Cancer in the General Population of Jeddah, Saudi Arabia: A Cross-Sectional Study
Source: Healthcare (Basel). 2026 Jun 17;14(12):1743. doi: 10.3390/healthcare14121743 (PMC13300078; doi:10.3390/healthcare14121743)
Supplement: Supplementary file 1 [file healthcare-14-01743-s001.zip › healthcare-4331695-Supplementary material 3 .pdf]

STROBE Statement—checklist of items that should be included in reports of observational studies

	Item No.	Recommendation	Page No.	Relevant text from manuscript
**Title and abstract**	1	(*a*) Indicate the study's design with a commonly used term in the title or the abstract	1	Title (page 1): "Awareness and Knowledge of Gastric Cancer in the General Population of Jeddah, Saudi Arabia: A Cross-Sectional Study."
		(*b*) Provide in the abstract an informative and balanced summary of what was done and what was found	1	Structured abstract on page 1 with Background, Objectives, Methods (cross-sectional survey of 1,400 adults in Jeddah, August-October 2025), Results, and Conclusions.
**Introduction**				
Background/rationale	2	Explain the scientific background and rationale for the investigation being reported	3	Section 1 (Introduction), page 3, lines 51-87: global and Saudi GC epidemiology, established risk factors, screening rationale in high-incidence countries (Japan and Korea), and summary of prior Saudi awareness studies.
Objectives	3	State specific objectives, including any prespecified hypotheses	4	Section 1 (Introduction), page 4, lines 97-103: three numbered study aims - (i) quantify public knowledge of GC across four predefined domains, (ii) identify specific knowledge gaps amenable to targeted interventions, and (iii) examine sociodemographic factors associated with knowledge.
**Methods**				
Study design	4	Present key elements of study design early in the paper	4	Section 2.1 (Study Design and Setting), page 4, lines 106-114: cross-sectional community survey with reporting per STROBE guidelines.
Setting	5	Describe the setting, locations, and relevant dates, including periods of recruitment, exposure, follow-up, and data collection	4	Section 2.1, page 4, lines 106-108: Jeddah, Saudi Arabia, with data collection between August and October 2025.
Participants	6	(*a*) *Cohort study*—Give the eligibility criteria, and the sources and methods of selection of participants. Describe methods of follow-up<br>*Case-control study*—Give the eligibility criteria, and the sources and methods of case ascertainment and control selection. Give the rationale for the choice of cases and	4	Section 2.2 (Sample and Recruitment), page 4, lines 116-121: 1,400 Jeddah residents recruited via convenience sampling at public malls and venues, supplemented by an electronic questionnaire distributed via social media. Inclusion criteria: age >=18 years, residence in Jeddah; no additional exclusion criteria.

		controls<br>*Cross-sectional study*—Give the eligibility criteria, and the sources and methods of selection of participants		
		(*b*) *Cohort study*—For matched studies, give matching criteria and number of exposed and unexposed<br>*Case-control study*—For matched studies, give matching criteria and the number of controls per case	N/A	Not applicable - this is a cross-sectional study without matching.
Variables	7	Clearly define all outcomes, exposures, predictors, potential confounders, and effect modifiers. Give diagnostic criteria, if applicable	5	Section 2.3 (Data Collection Instrument), page 5, lines 122-134: outcomes were knowledge scores across four domains (risk factors, symptoms and signs, prevention, management); predictors were eight demographic items (age, sex, education, occupation, personal GI history, personal GC history, family/friend GC history, source of health information).
Data sources/ measurement	8*	For each variable of interest, give sources of data and details of methods of assessment (measurement). Describe comparability of assessment methods if there is more than one group	5	Section 2.3, page 5, lines 122-134: 45-item structured questionnaire adapted from validated instruments used in prior GC awareness studies (refs. 19, 26); face and content validation by surgical faculty at King Abdulaziz University Hospital and additional expert clinicians; administered in Arabic with English back-translation; 37 knowledge items scored as true/false/don't know with one point per correct response (maximum score 37); pilot test in convenience sample (excluded from final analysis) confirmed item clarity and acceptability.
Bias	9	Describe any efforts to address potential sources of bias	5	Section 2.3, page 5, lines 122-134: bias-mitigation measures included expert content validation of the Arabic instrument, English back-translation to ensure linguistic fidelity, and pilot testing prior to full deployment. Residual sources of bias (sampling, response, recall) are discussed in the Strengths and Limitations subsection of the Discussion.
Study size	10	Explain how the study size was arrived at	4	Section 2.2, page 4, lines 116-121: a target sample of 1,400 adult residents was recruited consecutively until the target was reached. The sample size provides power to detect modest between-group differences in mean knowledge scores. Large sample size is noted as a study strength in the Strengths and Limitations subsection of the Discussion.

Continued on next page

Quantitative variables	11	Explain how quantitative variables were handled in the analyses. If applicable, describe which groupings were chosen and why	5	Section 2.4 (Statistical Analysis), page 5, lines 136-143: continuous variables presented as mean +/- SD; categorical variables as frequencies and percentages. Age was categorised into five groups (<=25, 26-35, 36-45, 46-55, >=56 years) and occupation into four groups (worker, non-worker, student, healthcare provider) for stratified analysis.
Statistical methods	12	(*a*) Describe all statistical methods, including those used to control for confounding	5	Section 2.4, page 5, lines 136-143: independent-samples t-test for two-group mean comparisons; one-way ANOVA with Tukey post-hoc for multi-group comparisons; chi-squared test for categorical associations; alpha = 0.05 (two-tailed); analyses performed in IBM SPSS Statistics. To identify factors independently associated with the total knowledge score, a multivariable linear regression model was fitted with the total score as the dependent variable and occupation, age group, educational attainment, sex, and family/friend history of GC as independent variables (Section 3.6, Table 5). Normality of the total score was confirmed (skewness and kurtosis within +/-1) before parametric testing.
		(*b*) Describe any methods used to examine subgroups and interactions	5-9	Subgroup analyses by occupation (Section 3, page 7-8, Table 2, Figure 3), age (Section 3, page 8-9, Table 3, Figure 4), sex, education, family history, and personal history of GC (Section 3, page 9, Table 4). Formal interaction tests were not conducted.
		(*c*) Explain how missing data were addressed	5	Section 2.2, 3.1, and 4.2: only fully completed questionnaires entered the final dataset (n = 1,400). The number of individuals approached was not recorded, so a formal response rate could not be calculated and non-response bias cannot be excluded; this is acknowledged as a limitation. Missing-data handling beyond this listwise approach was not required.
		(*d*) *Cohort study*—If applicable, explain how loss to follow-up was addressed<br>*Case-control study*—If applicable, explain how matching of cases and controls was addressed<br>*Cross-sectional study*—If applicable, describe analytical methods taking account of sampling strategy	5	Cross-sectional study: convenience sampling was used; analyses did not apply complex survey design weights, as no probability-based sampling frame was implemented.
		(*e*) Describe any sensitivity analyses	N/A	No sensitivity analyses were conducted.

**Results**				
Participants	13*	(a) Report numbers of individuals at each stage of study—eg numbers potentially eligible, examined for eligibility, confirmed eligible, included in the study, completing follow-up, and analysed	6	Section 3.1 (Participant Characteristics), page 6, lines 146-152: 1,400 respondents completed the survey and were included in the final analysis. Sample composition is detailed in Table 1 (page 6).
		(b) Give reasons for non-participation at each stage	N/A	Reasons for non-participation were not formally tracked at recruitment venues, given the convenience sampling design. This is acknowledged as a limitation in the Strengths and Limitations subsection of the Discussion.
		(c) Consider use of a flow diagram	N/A	A formal participant-flow diagram was not included; sample composition is fully described narratively in Section 3.1 and tabulated in Table 1.
Descriptive data	14*	(a) Give characteristics of study participants (eg demographic, clinical, social) and information on exposures and potential confounders	6	Section 3.1, page 6, lines 146-152, and Table 1: distributions of sex (53.8% women, 46.2% men), age groups, occupation (44.2% workers, 27.7% non-workers, 20.3% students, 7.8% healthcare providers), family/friend connection to GC (25.0%), and personal history of GC (1.8%).
		(b) Indicate number of participants with missing data for each variable of interest	N/A	All 1,400 included questionnaires were complete for the variables analysed; missing data per variable were therefore not separately reported.
		(c) *Cohort study*—Summarise follow-up time (eg, average and total amount)	N/A	Not applicable - this is a cross-sectional study.
Outcome data	15*	*Cohort study*—Report numbers of outcome events or summary measures over time	N/A	Not applicable - cohort-specific item.
		Case-control study—Report numbers in each exposure category, or summary measures of exposure	N/A	Not applicable - case-control-specific item.
		Cross-sectional study—Report numbers of outcome events or summary measures	6-7	Section 3.2 (Knowledge Scores by Domain), pages 6-7, lines 154-166: overall mean knowledge score 18.94 +/- 7.34 out of 37 (51.2%); domain-level scores - risk factors 4.95 +/- 2.04/11 (45.0%), symptoms and signs 4.54 +/- 2.87/10 (45.4%), prevention 4.91 +/- 2.09/10 (49.1%), management 2.99 +/- 1.95/6 (49.8%). Best-recognised items shown in Figure 2.
Main results	16	(*a*) Give unadjusted estimates and, if applicable, confounder-	7-9	Section 3, pages 7-9, Tables 2-4: unadjusted mean knowledge scores

	adjusted estimates and their precision (eg, 95% confidence interval). Make clear which confounders were adjusted for and why they were included		with SD presented for each subgroup, with P values from independent-samples t-tests, one-way ANOVA, and chi-squared tests as appropriate. Confounder-adjusted estimates are provided by the multivariable linear regression model (Section 3.6, Table 5), reporting unstandardized coefficients (B) with 95% confidence intervals and standardized coefficients (beta) for occupation, age group, educational attainment, sex, and family/friend history of GC.
	(*b*) Report category boundaries when continuous variables were categorized	5	Section 2.4: age categories (<=25, 26-35, 36-45, 46-55, >=56 years); occupation categories (worker, non-worker, student, healthcare provider); knowledge categories not used (continuous score retained).
	(*c*) If relevant, consider translating estimates of relative risk into absolute risk for a meaningful time period	N/A	Not applicable - the outcome is a continuous knowledge score rather than a risk estimate.

Continued on next page

Other analyses	17	Report other analyses done—eg analyses of subgroups and interactions, and sensitivity analyses	7-9	Section 3, pages 7-9: stratified analyses by occupation (Table 2, Figure 3), age (Table 3, Figure 4), sex, education, and personal/family connection to GC (Table 4); and a multivariable linear regression of factors independently associated with total knowledge score (Section 3.6, Table 5). No formal interaction or sensitivity analyses were performed; nominal p-values are reported without adjustment for multiple comparisons.
**Discussion**				
Key results	18	Summarise key results with reference to study objectives	10	Section 4 (Discussion), page 10, opening paragraph: four key findings - (i) overall knowledge was suboptimal (mean 51.2% of maximum); (ii) in multivariable analysis, educational attainment (beta = 0.30) and healthcare-provider occupation (beta = 0.23) were the strongest independent correlates of knowledge (both p < 0.001); (iii) older age (>=56 years) and family/friend connection to GC were independently associated with higher knowledge, while sex was not; (iv) risk factor and symptom recognition lagged behind prevention and management across all groups.
Limitations	19	Discuss limitations of the study, taking into account sources of potential bias or imprecision. Discuss both direction and magnitude of any potential bias	12	Section 4 (Discussion), Strengths and Limitations subsection, page 12: single-city sampling (Jeddah) with possible over-representation of higher socioeconomic and educational groups; under-representation of lower socioeconomic strata despite multi-channel recruitment; potential inflation of correct responses by guessing on true/false/don't know items; cross-sectional design precluding causal inference; absence of full psychometric testing (test-retest reliability, confirmatory factor analysis); limited granularity of H. pylori-specific items; multiple between-group comparisons without formal adjustment for multiple testing; response rate not calculable as the number approached was not recorded; no a priori power calculation; and potential social desirability bias inherent to self-reported knowledge surveys.
Interpretation	20	Give a cautious overall interpretation of results considering objectives, limitations, multiplicity of analyses, results from similar studies, and other relevant evidence	10-12	Section 4 (Discussion), pages 10-12: comparison with prior Saudi work (Alghamdi 2023, Alzahrani 2024, Jamal 2026, Alameer 2024); comparison with Iranian (Mansour-Ghanaei 2012) and UK (Adlard

				and Hume 2003) data; interpretation of the U-shaped age-knowledge relationship, including its attenuation after multivariable adjustment; international comparisons including the survival gap relative to high-incidence East Asian countries (Table 6). Multiplicity of subgroup comparisons is explicitly acknowledged.
Generalisability	21	Discuss the generalisability (external validity) of the study results	12	Section 4 (Discussion), Strengths and Limitations subsection, page 12: generalisability is constrained by single-city recruitment in Jeddah and the relatively higher socioeconomic and educational profile of the included sample; results may overestimate national-level GC awareness.
**Other information**				
Funding	22	Give the source of funding and the role of the funders for the present study and, if applicable, for the original study on which the present article is based	13	Funding statement, page 13: this project was funded by the KAU Endowment (WAQF) at King Abdulaziz University, Jeddah, Saudi Arabia. The funders had no role in study design, data collection, analysis, interpretation, or manuscript preparation.

*Give information separately for cases and controls in case-control studies and, if applicable, for exposed and unexposed groups in cohort and cross-sectional studies.

Note: An Explanation and Elaboration article discusses each checklist item and gives methodological background and published examples of transparent reporting. The STROBE checklist is best used in conjunction with this article (freely available on the Web sites of PLoS Medicine at http://www.plosmedicine.org/, Annals of Internal Medicine at http://www.annals.org/, and Epidemiology at http://www.epidem.com/). Information on the STROBE Initiative is available at www.strobe-statement.org.
