# Supplementary material for: Awareness and Knowledge of Gastric Cancer in the General Population of Jeddah, Saudi Arabia: A Cross-Sectional Study
Source: Healthcare (Basel). 2026 Jun 17;14(12):1743. doi: 10.3390/healthcare14121743 (PMC13300078; doi:10.3390/healthcare14121743)
Supplement: Supplementary file 1 [file healthcare-14-01743-s001.zip › healthcare-4331695-supplementary.pdf]

Supplementary Material 1: Standardized Recruitment Script

English Version:

Hello,

My name is [Your Name], and I am a medical student/research assistant at King Abdulaziz University. We are conducting a cross-sectional study to assess knowledge and awareness of gastric cancer among the general population in Jeddah, Saudi Arabia.

This study aims to identify gaps in public knowledge to help develop better education and early detection strategies. We are recruiting residents of Jeddah who are 18 years or older. Participation is completely voluntary, and you can withdraw at any time without any consequences.

Your responses will be kept strictly confidential and anonymous; no personal identifying information will be collected or shared. The questionnaire consists of 45 items and should take about 10-15 minutes to complete.

Do you have any questions about the study? Would you be interested in participating?

Thank you for your time and consideration.

Arabic Version (النسخة العربية):

مرحباً،

اسمي (اسمك}، وأنا طالب طب/مساعد بحثي في جامعة الملك عبد العزيز. نحن نقوم بدراسة مقطعية لتقييم المعرفة والوعي بسرطان المعدة بين السكان العامين في جدة، المملكة العربية السعودية.

تهدف هذه الدراسة إلى تحديد الفجوات في المعرفة العامة لمساعدة في تطوير استراتيجيات تعليمية أفضل واكتشاف مبكر. نحن نجند سكان جدة الذين تبلغ أعمارهم 18 عاماً أو أكثر. المشاركة طوعية تماماً، ويمكنك الانسحاب في أي وقت دون أي عواقب.

ستُحفظ إجاباتك بسرية تامة ومجهولة الهوية؛ لن يتم جمع أو مشاركة أي معلومات شخصية تعريفية. يتكون الاستبيان من 45 بنداً ويجب أن يستغرق حوالي 10-15 دقيقة لإكماله.

هل لديك أي أسئلة حول الدراسة؟ هل ترغب في المشاركة؟

شكراً لك على وقتك واهتمامك.

Supplementary Material 2

Full English Translation of the Gastric Cancer Knowledge and Awareness Questionnaire (45 items)

Correct answers are marked in BOLD for editorial/review purposes only. These marks were NOT visible to participants.

Demographic Information

1. Gender: □ Male □ Female
2. Age: ________ years
3. Educational level: □ Illiterate □ Primary/Intermediate/Secondary □ Diploma or higher □ Bachelor's degree or higher
4. Occupation: □ Unemployed/Non-worker □ Employed/Worker □ University student □ Physician or nurse (healthcare provider)
5. Family history of any gastrointestinal cancer: □ No □ Yes
6. History of gastrointestinal cancer among friends: □ No □ Yes
7. Do you currently have gastric cancer? □ No □ Yes

This questionnaire aims to measure the knowledge and awareness of Jeddah residents regarding gastric cancer and its management.

Risk Factors (11 items)

No.	Statement	Yes	No	Don't know
1	A diet rich in spicy and smoked foods can cause gastric cancer			
2	A diet containing nitrates (e.g., processed meats, hot dogs) can contribute to gastric cancer			
3	Freezing or canning foods with chemical preservatives is a risk factor for gastric cancer			
4	Cigarette smoking plays a role in gastric cancer			
5	Drinking very cold or very hot water plays a role in developing gastric cancer			
6	Alcohol consumption plays a role in gastric cancer			
7	Helicobacter pylori (bacterial) infection of the stomach is a risk factor for gastric cancer			
8	Family history of gastric cancer is a risk factor for developing gastric cancer			
9	Obesity/bariatric surgery increases the risk of gastric cancer			
10	Gastric cancer is one of the common lethal cancers in Saudi Arabia			

No.	Statement	Yes	No	Don't know
11	There are several types of gastric cancer			

Symptoms and Signs (10 items)

No.	Statement	Yes	No	Don't know
1	Unexplained weight loss is a symptom of gastric cancer			
2	Difficulty swallowing (dysphagia) is a symptom of gastric cancer			
3	Early satiety (feeling full quickly) is a symptom of gastric cancer			
4	Abdominal fullness/bloating is a symptom of gastric cancer			
5	Vomiting blood (hematemesis) is a symptom of gastric cancer			
6	Blood in stool (melena or hematochezia) can be a sign of gastric cancer			
7	Persistent nausea and vomiting are symptoms of gastric cancer			
8	Gastric cancer can cause anaemia			
9	Yellowing of the eyes (jaundice) is a symptom of gastric cancer			
10	Recurrent abdominal pain is a symptom of gastric cancer			

Prevention (10 items)

No.	Statement	Yes	No	Don't know
1	Fresh fruits and vegetables help prevent gastric cancer			
2	Antibiotics to treat H. pylori infection can prevent gastric cancer			
3	Quitting smoking helps prevent gastric cancer			
4	Quitting alcohol helps prevent gastric cancer			
5	Eating spicy and smoked foods reduces the risk of gastric cancer			
6	Preventing dental infections can help prevent gastric cancer			
7	Reducing consumption of frozen/preserved foods lowers the risk of gastric cancer			
8	If there is a family history of gastric cancer, first-degree relatives (parents, siblings) need regular follow-up and early screening			

No.	Statement	Yes	No	Don't know
9	Upper endoscopy (gastroscopy) is one of the most important methods for early detection of gastric cancer			
10	Early detection of gastric cancer significantly increases the chance of complete cure			

Management/Treatment (6 items)

No.	Statement	Yes	No	Don't know
1	Gastric cancer is treatable			
2	Surgery is used to treat gastric cancer			
3	Chemotherapy and radiotherapy are effective in treating gastric cancer			
4	A patient with gastric cancer can return to normal life after total gastrectomy			
5	Gastric cancer can be treated with laparoscopic (minimally invasive) surgery without open abdominal surgery			
6	Early-stage gastric cancer can be removed endoscopically through the mouth without conventional surgery			

Scoring: Each correct answer = 1 point; incorrect or "Don't know" = 0 points. Maximum total score = 37 points (9 risk factors + 10 symptoms + 11 prevention + 6 treatment items, excluding demographics).

Survey on Gastric Cancer Awareness in Jeddah

يهدف هذا الاستبيان الى قياس وعي ومعرفة مواطني مدينة جدة بسرطان المعدة وسبل علاجه

(سرطان المعدة)

المعلومات الديموغرافية :
الجنس ○ ذكر ○ انثى
العمر :
التعليم : ○ أمي ○ الابتدائية أوالثانوية ○ دبلوم أوأعلى ○ درجة البكالوريوس أو اعلى
الوظيفة : ○ غير موظف ○ موظف ○ طالب جامعي ○ طبيب أوممرض
هل هناك تاريخ لأي سرطان في الجهاز الهضمي في الأسرة: ○ لا ○ نعم
هل هناك تاريخ لأي سرطان في الجهاز الهضمي في الأصدقاء: ○ لا ○ نعم
هل تعاني من أورام المعدة : ○ لا ○ نعم

عوامل الخطر	نعم	لا	لا اعلم
1. النظام الغذائي الذي يحتوي على الأطعمة الحارة والمدخنة يمكن أن يسبب سرطان المعدة	○	○	○
2. النظام الغذائي الذي يحتوي على النترات مثل النقانق ، يمكن أن يكون لها دور في سرطان المعدة	○	○	○
3. يمكن أن يكون تجميد أو تعليب المواد الغذائية بالمواد الكيميائية عامل خطر للإصابة بسرطان المعدة	○	○	○
4. تدخين السجائر له دور في سرطان المعدة	○	○	○
5. شرب الماء البارد أو الحار له دور للإصابة بسرطان المعدة	○	○	○
6. شرب الكحول له دور في سرطان المعدة	○	○	○
7. عدوى المعدة البكتيرية هي أحد عوامل خطر الإصابة بسرطان المعدة	○	○	○
8. تاريخ العائلة المصابة بسرطان المعدة هو أحد عوامل الإصابة بسرطان المعدة	○	○	○
9- هل تعتبر عمليات السمنة من أخطار الإصابة سرطان المعدة	○	○	○
10- يعتبر سرطان المعدة من الأورام القاتلة المنتشرة بالسعودية	○	○	○
11- يوجد عدة أنواع من سرطان المعدة	○	○	○

أعراض وعلامات	نعم	لا	لا اعلم
1. فقدان الوزن هو أحد أعراض سرطان المعدة	○	○	○
2. عسر البلع هو أحد أعراض سرطان المعدة	○	○	○
3. الشبع المبكر هو عرض من أعراض سرطان المعدة	○	○	○
4. امتلاء البطن هو عرض من أعراض سرطان المعدة	○	○	○
5. الدم المصاحب للقيء هو أحد أعرض الإصابة بسرطان المعدة	○	○	○
6. الدم المصاحب للبراز هو أحد عوامل الخطر للإصابة بسرطان المعدة	○	○	○
7. الغثيان والقيء المتكرر من أعراض سرطان المعدة	○	○	○
8. سرطان المعدة يسبب فقر الدم	○	○	○
9- اصفرار لون العين هو أحد أعراض الإصابة بسرطان المعدة	○	○	○
10- الآم البطن المتكررة هو عرض من أعراض سرطان المعدة	○	○	○

الوقاية	نعم	لا	لا اعلم
1. الفواكه والخضروات الطازجة تمنع سرطان المعدة	O	O	O
2. المضادات الحيوية لعلاج عدوى معدية تمنع سرطان المعدة	O	O	O
3. الإقلاع عن التدخين يساعد في الوقاية من سرطان المعدة	O	O	O
4. الإقلاع عن الكحول يساعد في الوقاية من سرطان المعدة	O	O	O
5-. تناول الطعام الحار والمدخن يقلل من الإصابة بسرطان المعدة			
7. منع عدوى الأسنان يمكن أن يمنع سرطان المعدة	O	O	O
8. انخفاض استخدام الأغذية المجمدة يقلل من خطرالاصابة بسرطان المعدة	O	O	O
9. إذا كان هناك تاريخ عائلي من سرطان المعدة ، هناك حاجة إلى المتابعة والكشف المبكر في اقرباء المصاب من الدرجة الاولى(آب، ام، اخ، آخت)	O	O	O
10- يعتبر منظار المعدة من أهم وسائل الكشف المبكر عن سرطان المعدة	O	O	O
11- الكشف المبكر عن سرطان المعدة يزيد من نسبة الشفاء التام	O	O	O

العلاج	نعم	لا	لا اعلم
1. يوجد علاج لسرطان المعدة	O	O	O
2. تستخدم الجراحة لعلاج سرطان المعدة	O	O	O
3. العلاج الكيميائي والعلاج الإشعاعي فعال في علاج سرطان المعدة	O	O	O
4- يستطيع مريض سرطان المعدة أن يمارس حياته الطبيعة بعد استئصال الكامل للمعدة	O	O	O
5- يمكن علاج سرطان المعدة بتقنية المناظير الجراحية بدون الحاجة لفتح البطن	O	O	O
6- يمكن استئصال سرطان المعدة في المراحل المبكرة بتقنية منظار الفم بدون الحاجة للجراحة	O	O	O
